# Barriers and missed opportunities in PrEP uptake, use and care among men who have sex with men with recent HIV infection in the Netherlands

**DOI:** 10.1371/journal.pone.0310621

**Published:** 2025-01-06

**Authors:** Jeffrey C. D. Koole, Maarten R. D. Bedert, Feline de la Court, Irene Bais, Ferdinand Wit, Janneke Stalenhoef, Tania Mudrikova, Katalin Pogany, Birgit van Benthem, Maria Prins, Udi Davidovich, Marc van der Valk

**Affiliations:** 1 Department of Infectious Diseases, Public Health Service of Amsterdam, Amsterdam, The Netherlands; 2 Amsterdam University Medical Centers, Department of Infectious Diseases, Amsterdam Infection and Immunity Institute, University of Amsterdam, Amsterdam, The Netherlands; 3 HIV Monitoring Foundation, Amsterdam, The Netherlands; 4 Department of Social Psychology, University of Amsterdam, Amsterdam, The Netherlands; 5 Department of Internal Medicine, OLVG, Amsterdam, The Netherlands; 6 Department of Internal Medicine and Infectious Diseases, University Medical Center Utrecht, Utrecht, The Netherlands; 7 Department of Internal Medicine, Maasstad Hospital, Rotterdam, The Netherlands; 8 Center for Infectious Disease Control, National Institute for Public Health and the Environment (RIVM), Bilthoven, The Netherlands; HIV/STI Surveillance Research Center and WHO Collaborating Center for HIV Surveillance, Institute for Future Studies in Health, Kerman University of Medical Sciences, ISLAMIC REPUBLIC OF IRAN

## Abstract

**Introduction:**

Oral pre-exposure prophylaxis (PrEP) prevents Human Immunodeficiency Virus (HIV) acquisition. In the Netherlands, PrEP is accessible through the national PrEP program (NPP) or general practitioners (GP). Still, some men who have sex with men (MSM) entering HIV care indicated having PrEP experience prior to diagnosis. We aimed to identify barriers and missed opportunities in PrEP uptake, care and use among MSM with HIV and previous PrEP experience.

**Methods:**

Between March 2022-March 2023, we conducted semi-structured in-depth interviews on PrEP among MSM diagnosed with HIV from 2019 onwards with previous PrEP experience. Interviewees were recruited through their HIV treatment centers and social media.

**Results:**

Of the 11 included MSM, most reported significant PrEP-uptake delay because of the limited NPP capacity and high threshold of accessing PrEP from GPs (e.g. stigma, lack of sexual health expertise). Additional uptake or use barriers included anticipated/experienced side-effects, burden of daily pill-taking or event-driven regimen complexity, the latter leading to PrEP discontinuation. Missed opportunities in counseling on adherence and safer sex alternatives after discontinuation were reported. Most interviewees considered informal PrEP unsuitable.

**Conclusion:**

PrEP uptake delay played a crucial role in context of HIV infection among MSM with HIV and previous PrEP experience. HIV diagnoses at or shortly after PrEP initiation emphasize the importance of ensuring rapid and timely PrEP access. Uptake barriers at GPs, stigma on sexuality, lack of expertise, and missed care opportunities need to be addressed. Early detection of PrEP protocol/user-mismatch and counseling on safer sex alternatives after discontinuation are pivotal for sustainable HIV prevention.

## Introduction

Oral HIV pre-exposure prophylaxis (PrEP) prevents HIV acquisition safely and effectively and current regimens include daily or event-driven use (2 pills 2–24 hours before sex, 1 pill 24 hours and 1 pill 48 hours after the first dose (2-1-1), also referred to as intermittent or on-demand regimen) [1, 2]. Since 2015, World Health Organization (WHO) has recommended PrEP use for HIV key populations [3]. Following Dutch national guidelines, PrEP eligible individuals include men who have sex with men (MSM) who recently had condomless anal sex with a sexual partner with either unknown HIV status or (presumed) detectable HIV viral load, rectal bacterial sexually transmitted infection (STI) or syphilis, or HIV post-exposure prophylaxis (PEP) use [4]. In the Netherlands, since 2019, formal PrEP has been accessible through the Dutch national PrEP pilot program (NPP) at Centers for Sexual Health (CSH), or general practitioners (GPs) [5]. The NPP is a governmentally subsidized pilot from 2019–2024 with a capacity of 8,500 individuals [5]. PrEP and PrEP care is provided through the National PrEP Program by healthcare professionals at all CSHs in the Netherlands, and funded by the Dutch Ministry of Health. High PrEP demand exceeding the progamme’s capacity resulted in long NPP waitlists [5]. Nation-wide, between Augustus and October 2023, the NPP waitlist included 2,000–2,600 individuals (personal communication Dutch National Institute for Public Health and the Environment on 2 November 2023). Before the NPP, formal PrEP was very limitedly available, as of 2015, for Amsterdam PrEP demonstration project participants [6].

Despite PrEP availability and effectiveness, some MSM who were recently diagnosed with HIV in the Netherlands reported having PrEP experiences prior to diagnosis. HIV monitoring foundation [Stichting hiv monitoring (SHM)] collects national data on people with HIV (PWH) entering care. Between 2018 and 2022, PrEP use data were available for 992/2,926 (33.9%) PWH. Of those, 106 (10.7%) indicated PrEP use prior to their HIV diagnosis, of which 97 were MSM [7]. A similar picture emerges from the United States, where over 25% of newly HIV diagnosed MSM reported past PrEP use, and median time between PrEP discontinuation and HIV diagnosis was less than 6 months [8] (1)(1)(Cannon, Ramchandani, Buskin, Dombrowski, & Golden, 2022). These findings raise concerns about PrEP access, care and use efficacy. In this qualitative study, we aimed to identify barriers and missed opportunities in PrEP uptake, care and use among MSM with HIV and previous PrEP experience who were diagnosed while formal PrEP was readily available in the Netherlands (from 2019 onwards).

## Methods

### Study design

We collected data using semi-structured in-depth interviews (IDIs) on PrEP knowledge, uptake, care, use and discontinuation, based on recent SHM data and available literature.

### Interviewees and recruitment

Prospective national data on PWH have been collected through the Dutch AIDS Therapy Evaluation in the Netherlands (ATHENA) national observation HIV cohort, maintained by SHM [9, 10]. About 98% of PWH entering care in one of the 24 HIV treatment centers in the Netherlands provided informed consent for ATHENA participation. MSM diagnosed with HIV from 2019 onwards with previous PrEP experience were eligible for this study participation. PrEP experience was defined as: attempting to access PrEP through the NPP, GPs or other prescribing physicians (e.g., internal medicine specialist), and formal or informal PrEP use. Informal PrEP, also referred to as off-label or non-prescribed PrEP, includes pills imported from abroad, ordered online or obtained through sexual/social networks, and lacks formal PrEP care aspects (e.g., HIV testing before initiation and during use) [11, 12]. Potential interviewees were identified using purposeful sampling using SHM data, and recruited through their respective HIV treatment centers (i.e., OLVG, Amsterdam; University Medical Center Utrecht (UMCU), Utrecht; Amsterdam University Medical Center (Amsterdam UMC), Amsterdam; Maasstad Hospital, Rotterdam; Diagnostic Center (DC) Clinics, Amsterdam). Additional recruitment took place via social media (e.g., advertisements designed for and by MSM on popular MSM community pages), and the mobile dating application Grindr. Written informed consent was obtained prior to inclusion. Interviewees received a financial compensation (a 25 euro valued gift card).

### Data collection and analysis

Between March 2022 and March 2023, IDIs were conducted in Dutch or English, in person (n = 9) or by video conferencing or phone (n = 2), as preferred by the interviewee. IDIs were audio-recorded, transcribed verbatim, and analyzed using open and axial coding using MAXQDA Plus 2022 (Release 22.0.0) software by three researchers. Axial coding was used to identify relations between themes and distil categories until consensus was reached within the research team. Analysis was ongoing during recruitment. A constant comparative approach was used by iteration of data collection and analysis. Data collection continued until saturation was reached. PrEP uptake delay was defined as the time between attempting to access PrEP, and either PrEP intake consultation or initiation, whichever came first.

### Ethics

Medical Ethics Committee of the Amsterdam UMC, location Academic Medical Center (AMC) granted an exemption from further assessment of the submitted protocol for this study (reference number: W21_456 # 21.506) considering the Medical Research Involving Human Subjects Act. The ATHENA cohort was approved by the institutional review board of all participating centers. PWH entering care provided verbal informed consent or opted-out for ATHENA participation. ATHENA data are pseudonymized before being provided to investigators and may be used for scientific purposes. A designated quality management coordinator safeguards compliance with European General Data Protection Regulation [9]. Additional written informed consent was obtained prior to participation in this qualitative study.

## Results

### Interviewee characteristics

We included 11 interviewees, aged between 21–67 years, of whom seven (64%) born in the Netherlands, five (45%) had a college degree or higher, and nine (82%) were either employed or student. Table 1 displays the interviewee characteristics on socio-demographics, HIV diagnosis and PrEP access, care and use (Table 1). Interviewees accessed PrEP informally (n = 2) and formally (n = 9). Both informal PrEP users were not HIV tested prior to initiation and were diagnosed with HIV 1 and 6 months thereafter, of whom one presumably acquired HIV several years before initiation based on reported clinical symptoms (multiple suspected episodes of *Varicella zoster virus* infection 3–4 years prior to initiation). All 9 who accessed formal PrEP were offered HIV testing before initiation. One opted out for HIV testing during his PrEP intake consultation at a GP abroad, experienced severe diarrhea and weight loss (acute HIV infection symptoms) shortly after initiation, and self-tested HIV-positive after 3 months of use. Three tested positive at PrEP intake consultation, precluding them from starting PrEP. Five started PrEP after being tested HIV-negative. Among these five formal PrEP users, two were diagnosed with HIV 1 and 3 months after initiation, respectively. Both most likely acquired HIV shortly before PrEP initiation, according to their HIV treating physician. One formal PrEP user was diagnosed with HIV after 2 years of daily use with self-reported good adherence. Neither CSH healthcare professionals nor his HIV treating physician could provide a clear explanation for his HIV acquisition. One tested positive 4 months after PrEP initiation and incorrect use of event-driven regimen. One discontinued PrEP because he considered both regimens unsuitable, and was diagnosed with HIV a few months thereafter. Table 2 provides an overview of interviewee quotations, presented as transcript number and position (Table 2).

**Table 1:** PrEP uptake, care and use, socio-demographic and HIV diagnosis characteristics of 11 MSM with HIV and previous PrEP experience in the Netherlands.

#	Age, years	COB	Sexual behavior group	PrEP uptake, care, use and discontinuation									First positive HIV test result (HIV diagnosis)		Estimated moment of HIV infection
				Uptake route	Country of uptake	Preferred uptake route	Uptake delay^a^, months	HIV test result before initiation	Regimen used	Use duration, months	Regimen adherence^f^	Reason to discontinue	Moment	Location	
1	30–40	NL	MSM	Sexual network (I)	NL	CSH	A few	Not performed	ED	6	Good	Running out of informal pills	2 months after PrEP dinscontinuation	CSH	1 month after PrEP discontinuation
2	40–50	NL	MSM	IMS (F)	N/a	N/a	N/a	Positive^c^	N/a	N/a	N/a	N/a	At PrEP intake consultation	IMS	Before PrEP initiation
3	40–50	BR	MSM	Sexual network (I)	NL	N/a	N/a	Not performed	ED	1	Good	Positive HIV test result	1 month after PrEP initiation	CSH	4 years before PrEP initiation
4	50–60	NL	MSM	CSH (F)	NL	CSH	12	Negative^d^	ED	1	Good	Positive HIV test result	1 month after PrEP initiation	CSH	Before PrEP initiation
5	60–70	NL	MSM	CSH (F)	NL	CSH	12	Negative	Daily	24	Good	Positive HIV test result	24 months after PrEP initiation	CSH	During PrEP use
6	20–30	BR	MSM	GP (F)	SA	CSH	2^b^	Not performed^e^	Daily	3	Good	Positive HIV test result	3 months after PrEP initiation	Self-test at home	Before or shortly after PrEP initiation
7	20–30	SU	MSM	GP (F)	NL	CSH	N/a	Negative	Daily, ED	4	Not good	Positive HIV test result	4 months after PrEP initiation	CSH	During PrEP use
8	60–70	NL	MSM	GP (F)	NL	GP	N/a	Negative	ED	6–7	Good	Burden of daily pill-taking, burden of complexity of ED regimen	A few months after PrEP discontinuation	GP	After PrEP discontinuation
9	30–40	BR	MSM	GP (F)	NL	CSH	12	Negative^d^	Daily	3	Good	Positive HIV test result	3 months after PrEP initiation	GP	Before PrEP start (HIV diagnostic window period)
10	50–60	NL	MSM	GP (F)	NL	CSH	18	Positive^c^	N/a	N/a	N/a	N/a	At PrEP intake consultation	GP	Before PrEP initiation
11	30–40	NL	MSM	CSH (F)	NL	CSH	3	Positive^c^	N/a	N/a	N/a	N/a	At PrEP intake consultation	CSH	<1 month before PrEP initiation

#: interviewee number, BR: Brazil, COB: Country of birth, CSH: center for sexual health, ED: event-driven, F: formal PrEP, GP: general practitioner, I: informal PrEP, IMS: internal medicine specialist, MSM: men who have sex with men, N/a: not applicable, NL: the Netherlands, NPP: national PrEP pilot program, PrEP: HIV pre-exposure prophylaxis, SA: South-Africa, SU: Suriname

^a^: Time between *attempting to access PrEP* and either *PrEP intake consultation* or *starting PrEP*, whichever came first

: he postponed his PrEP pursuit for 2 years because formal PrEP was not yet available

^c^: those having a first positive HIV test result at PrEP intake consultation did not start PrEP

^d^: presumed false-negative because of HIV diagnostic window period

^e^: he had a negative HIV self-test result 1 month before PrEP initiation and decided to opt out for HIV testing before initiation

^f^: self-reported

**Table 2:** Barriers and missed opportunities in PrEP uptake, care, and use among 11 MSM with HIV and previous PrEP experience in the Netherlands: Interviewee quotations.

Transcript 9, Pos. 4	“Through a conversation with a friend, who advised me to start using it [PrEP]. […] I talked about it with that friend, and he said, ’Maybe it’s a good idea to start using PrEP.’ Well, that seemed like a good idea to me too.“
Transcript 2, Pos. 6–8)	“In 2015, I started dating men, you know. And yeah, it [PrEP] was mainly through word of mouth by people who were undetectable, and also people who used PrEP to prevent HIV. "On PrEP" was written in their profile, and then you start googling to find out what that is. I think that’s how I learned about it too.”
Transcript 12, Pos. 4–6	"I was in Asia in 2015, and by then I had already heard about it [PrEP] because many people were bringing it from Thailand. And I thought that if I wanted to use it [PrEP], I had an easy way now.”
Transcript 5, Pos. 15	“There was an item in the newspaper about a PrEP study in Amsterdam. I thought: “What is that?” Then you delve into it and explore it yourself."
Transcript 8, Pos. 58–63	“I went to the CSH for HIV/STI testing. They looked at me really concerned, like: "What are you doing here?". So, yeah, I think it was just out of concern that they wanted to bring it [PrEP] up. So, I had a conversation with someone who told me more about it [PrEP]."
Transcript 1, Pos. 62–64)	“Yes, I absolutely prefer getting PrEP through the CSH. First of all, because I had good experiences there. The understanding and knowledge were, I think, the most organized and up-to-date there. Also, when I go to the CSH, I don’t have to go to the general practitioner, even though I had a good relationship with him.”
Transcript 11, Pos. 52–53, 115	"About two years ago at the CSH, they [healthcare professionals] brought it [PrEP] up again. […] So, I decided to register for it [NPP], but they immediately informed me that it was fully booked, and there might be a long waiting period. […] I think I was registered for about a year and a half when I decided to access it [PrEP] through my GP."
Transcript 10, Pos. 35, 88–89	"There were two options: I could either go to my GP and request PrEP from them, or I could go to the CSH. In the end. I initially tried accessing PrEP [through the NPP] at the CSH, but there was a queue. After that, I switched GPs, and with my new GP, I had a little more interaction, so I asked [my GP] for PrEP. There was about one year between the moment I tried to access PrEP at the CSH and taking my first [PrEP] pill."
Transcript 4, Pos. 48	“I went to my GP a few times when I couldn’t go to the CSH for an STI test. But yeah, she always indicated that she didn’t have much knowledge about it [sexual health]. So, I didn’t trust to go to the GP for these kinds of things. One time, I just wanted to do an STI test like I normally do four times a year, but she [GP] simply refused because she was afraid of making a wrong diagnosis.”
Transcript 6, Pos. 18	“During the [COVID-19] pandemic, it was very awkward, I had symptoms of gonorrhea and monkeypox. And every time she [GP] said: “Shouldn’t you use a condom?” Yes, I felt judged. It seemed so easy for a 50-year-old straight female to tell people things like that.”
Transcript 10, Pos. 35, 42–43	"I could not imagine discussing PrEP or sexual health with my previous GP. I think I visited that GP once, and then I switched to the second GP who was recommended by a friend of mine. This second GP had a lot of expertise and a lot of gay clients, so it was easier to discuss these matters [PrEP and sexual health] with him, and I asked him for PrEP.”
Transcript 8, Pos. 6, 77–79, 113, 129–133	The [NPP] waitlist was really long there [CSH]. They [CSH healthcare professionals] said that I could also go to the GP. So, I asked my GP for PrEP and he explained how it worked: “You can take it every day or around the sex.” […] Okay, so, I used it [PrEP] every day for one week because every time I took it, I got really severe headaches. Because my partner and I didn’t see each other that often, I though I’ll just take it around the sex, that’s better because I couldn’t tolerate it [headaches]. […] As I have been told by my HIV treating physician, I did not take it [event-driven regimen] properly, in terms of the dosages. The information I received from my GP was: one instead of two pills two hours before [sex], then one pill afterwards, and the next day one more pill [i.e., 1-1-1 instead of 2-1-1].”
Transcript 12, Pos. 18, 22	“It [PrEP] can cause side-effects. My mother has kidney failure, so well, it is also known that it [PrEP] can have an impact on your kidneys, so I was extra cautious, because it [kidney problems] might be familiar. […] I also noticed people around me were using PrEP, and they were doing well with it.”
Transcript 9, Pos. 175–177	“A few years ago, I had hepatitis B [virus infection] for which I had to take daily medication for several years. It was an unattractive idea to take it [PrEP] every day, so I just took it before and after sex. But that wasn’t easy either, because I usually had sex through dating sites where they [sex partners] wanted to come over in an hour, while you had to take that PrEP two hours in advance. […] It also occurred a few times that I took that pill in advance, but eventually the other person didn’t show up. […] After all, it was less of a fuss to use a condom than taking PrEP.”
Transcript 12, Pos. 68–71	“I wouldn’t take PrEP from friends. Well, because I want my health to be well monitored, and with that kidney test and all, I consider that important. So, I would rather consider getting PrEP from my GP.”
Transcript 6, Pos. 192	“Some friends took PrEP from India; a few super rich people could afford that. One guy told me he used PrEP from India. I thought it could be fake, like vitamin C, so I didn’t trust it.”
Transcript 1, Pos. 54–8	“It gave a certain shame, like, I didn’t dare to ask for it [PrEP]. […] I could not enter the PrEP program here [NPP at the CSH] because there were no available spots left. Later, additional spots became available, but by then it was already too late to enter the program. There was also a double-blind PrEP study, but there were no spots left either. So, eventually I went to the general practitioner myself, I pushed myself to do so. Fortunately, my GP was aware of PrEP and willing to help me. But then I coincidentally came into contact with someone I had met before who happened to have spare PrEP bottles at home. He still owed me some money, and in exchange, he gave me those bottles for a few months.”

### MSM community as main PrEP information source

Rather than official or formal information sources, interviewees mainly gained and exchanged PrEP knowledge through conversations within their social or sexual networks (MSM community) rather than official or formal information sources (Transcript 9, Pos. 4). Participants encountered PrEP through online dating app profiles, such as Grindr (Transcript 2, Pos. 6–8). Some interviewees were already aware of PrEP before it became formally available in the Netherlands, and one was introduced to informal PrEP by his social network abroad (Transcript 12, Pos. 4–6). Nevertheless, some interviewees encountered basic PrEP information through news items or during sexual health consultations, and gathered more information about it themselves afterwards (Transcript 5, Pos. 15; Transcript 8, Pos. 58–63).

### Limited national PrEP program capacity delayed PrEP uptake

Interviewees were aware of the different PrEP uptake routes, and most reported the NPP as their preferred route because of comfortability, familiarity and expertise (Transcript 1, Pos. 62–64). Still, PrEP uptake was considerably delayed because of the limited capacity of the NPP. Table 3 shows an overview of the identified barriers and missed opportunities in PrEP uptake, care and use (Table 3). Interviewees reported their (unsuccessful) NPP enrolment attempts (Transcript 11, Pos. 52–53, 115). Some accessed PrEP with over one year of uptake delay, and were diagnosed with HIV at or shortly after PrEP intake consultation. One interviewee reported accessing PrEP at his GP after 12 months of delay, and was diagnosed with HIV 3 months after initiation (Transcript 10, Pos. 35, 88–89). According to his HIV treating physician, he tested false negative at PrEP intake consultation because of the HIV diagnostic window period.

**Table 3:** Barriers and missed opportunities in PrEP uptake, care, and use among 11 MSM with HIV and previous PrEP experience in the Netherlands.

**PrEP uptake barriers** 1. Limited NPP capacity 2. High threshold for requesting PrEP from GPs a) Stigma on sexuality and sexual behavior b) Fearing judgment because of sexual behavior c) Lack of sexual health or PrEP expertise d) High financial costs of regular HIV/STI testing 3. Need of parental approval for using PrEP as being a minor 4. Fearing side-effects (allergic reaction, kidney problems) 5. Not considering informal PrEP an option a) Lack of standard care (HIV testing before initiation and during use, side-effects monitoring) b) High costs of regular HIV/STI testing c) High costs of informal pills d) Doubting the legitimacy of informal pills
**PrEP care missed opportunities** 1. Lack of counseling on adherence (instructions on correct pill-taking; safely switching, discontinuing and restarting regimens) 2. Lack of counseling on practicing safer sex alternatives after discontinuation 3. Lack of standard care when using informal PrEP
**PrEP use barriers** 1. Experiencing side-effects (severe headaches during daily regimen use) 2. PrEP protocol/user-mismatch a. Burden of daily-pill taking b. Burden of complexity of event-driven regimen

CSH: Centre of Sexual Health, GP: general practitioner, NL: the Netherlands, NPP: national PrEP pilot program, PrEP: HIV pre-exposure prophylaxis, STI: sexually transmitted infections

### High threshold at general practitioners delayed PrEP uptake

Interviewees experienced a high threshold for requesting PrEP from GPs because of anticipated discomfort with discussing sexuality or sexual health, and due to anticipated stigma or fearing being judged. Anticipated or actual lack of sexual health and PrEP expertise among GPs was reported, and as a result, some interviewees decided to access PrEP through other routes. One interviewee who reported uptake barriers at his GP, eventually accessed PrEP through the NPP after one year of delay, and was diagnosed with HIV at his PrEP intake consultation (Transcript 4, Pos. 48). PrEP uptake was delayed or withheld because of the type of relationship some interviewees had with their GP, such as discomfort with discussing sexual health or anticipated/experienced stigma. This was a fundamental concern as they feared being judged for their sexual behavior, including sexualized drug use (Transcript 6, Pos. 18). One interviewee experienced high threshold requesting PrEP from his GP, and was only able to request it after he switched to another GP (Transcript 10, Pos. 35, 42–43). Another barrier for obtaining PrEP at GPs was the higher financial costs compared to the lower subsidized costs of PrEP care at the NPP. Being a sexually active adolescent MSM exacerbated the impact of the above barriers as described by the following interviewee: he first postponed his PrEP pursuit because of needing parental approval to approach his GP, and when he became of age, PrEP uptake was delayed because of the limited NPP capacity. After receiving multiple STI notifications from his sexual partner, he requested and accessed PrEP through his GP, and started daily use. Because of side-effects (severe headaches), he switched to an event-driven regimen within the first month of use. He was diagnosed with HIV 4 months after initiation. In hindsight, he reported incorrect use of event-driven regimen because of receiving incorrect pill-taking instructions from his GP (Transcript 8, Pos. 6, 77–79, 113, 129–133).

### Fearing side-effects delayed PrEP uptake

Interviewees reported that fearing PrEP side-effects postponed their PrEP pursuit. One interviewee with a close family member with kidney problems initially postponed his PrEP pursuit because of worrying about renal side-effects (Transcript 12, Pos. 18, 22). After 3 months of delay, he accessed PrEP through the NPP at CSH and was diagnosed with acute HIV infection at his PrEP intake consultation.

### Missed PrEP care opportunities and barriers during PrEP use

Most interviewees who accessed formal PrEP did not attribute their subsequent HIV diagnosis to the level of PrEP care provided by CSHs and GPs. They reported being well-informed about the pros and cons of the different regimens (counseling), and were able to motivate their choice of regimen in line with their needs and preferences. Still, one interviewee reported a mismatch between current available regimens and his needs and preferences (PrEP protocol/user-mismatch). He discontinued event-driven use because he experienced this regimen as complex and its related planning aspects as too burdensome. He did not switch to daily regimen because of the burden of daily pill-taking. As a result, he discontinued PrEP, reported switching back to condom-use but was diagnosed with HIV two months afterwards (Transcript 9, Pos. 175–177).

### Informal PrEP not considered an option

Most did not consider informal PrEP a suitable option because it lacks care. Monitoring potential side-effects during use was considered an important PrEP care aspect (Transcript 12, Pos. 68–71). Also, high costs of online offered informal pills, and doubting their legitimacy were reasons not to consider informal PrEP as an option (Transcript 6, Pos. 192). Still, some used informal PrEP in order to avoid requesting PrEP from their GP, or considered it a temporary solution until they could access formal PrEP while being on the NPP waitlist (Transcript 1, Pos. 54–8). One interviewee used informal PrEP, ran out of his pills after 6 months of use, discontinued PrEP and was diagnosed with HIV 2 months later.

## Discussion

In this qualitative study of barriers to PrEP uptake, care and use among MSM recently diagnosed with HIV despite past PrEP experience, we found that formal PrEP uptake was considerably delayed, which was possibly instrumental in the context of their HIV acquisition. Most were diagnosed with HIV at PrEP intake consultation or shortly after PrEP initiation, emphasizing the importance of rapid and timely access to formal PrEP. PrEP use is associated with engaging in sexual behavior that can lead to the acquisition of HIV/STI, including condomless anal sex, sexualized or injecting drug use and engaging in group sex, [13] and often requested at times of increased perceived vulnerability to HIV acquisition [14]. New HIV diagnosis at or shortly after PrEP initiation emphasizes the importance of ensuring rapid and timely access to PrEP.Our findings are supported by quantitative reports from the Netherlands: The yearly monitoring report from the ATHENA cohort [7], cshows that, up until December 31, 2023, 51 individuals tested HIV-positive at PrEP screening, while four individuals seroconverted while on the NPP waiting list and 65 indicated that they wanted to use PrEP but had no access to it. Given the declining number of new HIV seroconversions over the recent years, these findings indicate that MSM with HIV and PrEP experience might be driving factors of the ongoing HIV epidemic in the Netherlands.

We identified several barriers delaying formal PrEP uptake. Most interviewees could not (timely) access PrEP through the NPP at CSHs, even though this was their preferred uptake route because of comfortability, familiarity and sexual health expertise. As a consequence, some were diagnosed with HIV at PrEP intake, which corresponds with earlier reported quantitative findings on PrEP barriers [15]. In September 2023, the Dutch government decided to continue subsidising formal PrEP and its care through the NPP at CSHs after its initial end date, August 2024 [16, 17]. Nonetheless, NPP continuation alone does not guarantee its rapid and timely access. NPP waitlists are likely to remain or even increase because of high PrEP demand and barriers for accessing PrEP through GPs. Moreover, new NPP uptake barriers may arise, such as increased financial costs as governmental PrEP subsidies are planned to be reduced for both its users and the healthcare providers. Our findings illustrate the importance that the NPP and CSHs make necessary efforts to ensure PrEP accessibility in a low-threshold manner avoiding the formation of waitlists or procedural delay. Failing to provide services in such a manner is likely to result in HIV infections that could have been averted, also referred to as “waitlist infections”. Such infections, which are typically transmitted during the acute phase of the infection, are considered important instigators of ongoing HIV transmission.

For those who could not access PrEP through the NPP, accessing PrEP through GPs was no easy alternative because of stigma on sexuality, fearing judgment, discomfort with discussing sexual health, or anticipated/experienced lack of sexual health or PrEP expertise among GPs. This corresponds with earlier findings on perceived healthcare discrimination among MSM [18], and with earlier qualitative research in the Netherlands, prior to the NPP rollout in 2019, showing that some GPs were unable or unwilling to support the needs of MSM using informal PrEP [19]. Similar concerns were found regarding HIV testing and sexual health services at GPs linked to late HIV diagnoses in the Netherlands [20, 21]. GPs need to become more accessible for MSM and other sexual minorities by improving sexual health expertise and attitudes towards such key populations, providing a more comfortable setting to discuss sexuality.

For those who could not access formal PrEP, informal PrEP was a poor option. Few considered informal PrEP as temporary alternative while being on the NPP waitlist. Since informal PrEP lacks care, its users may not get HIV tested before initiation and may have undiagnosed HIV, as was the case for one interviewee. This can lead to HIV strains containing PrEP resistance-associated mutations (RAMs), which limits treatment options. PrEP RAMs were detected in 9 (19.6%) of 46 MSM with recent HIV who indicated PrEP use prior to diagnosis and with genotypic resistance data available [10]. In our study, genotypic resistance data were not available. One interviewee was diagnosed with HIV despite self-reported good adherence to daily regimen, also referred to as PrEP failure. PrEP failure is rare and only few cases have been reported in the Netherlands [22, 23].

### Importance of more tailored PrEP care

Although PrEP care was highly valued by interviewees who received it, we identified several missed care opportunities regarding PrEP side-effects perceptions and sexual behaviour post PrEP cessation. It was important for our participants to address concerns regarding adherence and side-effects before and after PrEP initiation, and receive assistance with management of safer sex alternatives after PrEP discontinuation (e.g., condom use, viral load sorting, also referred to as undetectable equals untransmittable or U = U). But most importantly, we identified the need for better tailored counseling on regimen choice and its experienced burden, and revisiting the latter as PrEP use progresses. Prior experiences with long-term oral medication, changes in sexual behavior and perceived chances of HIV/STI acquisition can lead to regimen switching, discontinuation or reverting to HIV prevention strategies other than PrEP [24]. Regimen switching is prone to experiencing user difficulties (e.g., side-effects, adherence difficulties, PrEP protocol/user-mismatch) but also provides care opportunities in the form of tailored counseling [25, 26]. Recent qualitative findings among Belgian PrEP providers on PrEP care quality showed that client-centered care, care continuity and more comprehensive care package (e.g., STI management, mental health counseling, expertise on interplay between PrEP, behavior, mental well-being and substance use) were important and helpful themes. Additionally, counseling on novel HIV prevention strategies, such as bi-monthly injectable cabotegravir as PrEP [27, 28], should be considered when these have become available in the Netherlands.

In sum, making sure MSM have early access to formal PrEP and tailored PrEP care is a crucial step in getting to zero new HIV infections and ending the HIV and AIDS epidemic as a public health threat by 2030.

### Study strengths and limitations

Our study has several limitations. Our IDIs included discussing one’s HIV diagnosis and sexual behavior, which might have led to a selection bias of those feeling comfortable discussing these topics. Also, the sensitive nature of the discussed topics might have led to social desirability bias. Furthermore, because PrEP landscapes in terms of eligibility, access and care modalities rapidly evolve, our findings are limited to the time and setting of data collection.

Our study has several important strengths. Reaching PrEP users who have seroconverted post-PrEP-use or closely prior to PrEP initiation is a daunting task in settings of very low HIV incidence rate, such as the Netherlands. The availability of relevant nation-wide data of people with HIV answering to these profiles and the possibility to recruit these participants through their HIV treatment centers reduced chances of selection bias and increased its effectiveness. Despite the fact that the number of potential participants was small due to very specific eligibility criteria, this qualitative investigation, by highly-trained interviewers, managed to reveal complex trajectories of missed PrEP opportunities that would not have not been captured through quantitative methods. Such data can contribute to better understanding of current barriers for PrEP uptake and expose niche needs of future potential PrEP users in the Netherlands.

## Conclusions

Among MSM with HIV and previous PrEP experiences, PrEP uptake delay was important in the context of HIV diagnosis. Uptake was delayed because of limited national PrEP program capacity and barriers for accessing PrEP through general practitioners. New HIV diagnosis at or shortly after PrEP initiation point at the importance of ensuring rapid and timely PrEP access. Additionally, uptake barriers at general practitioners, such as lack of expertise and stigma on sexuality, need to be addressed. Counselling on side-effects concerns and safer sex alternatives after PrEP discontinuation as well as early detection of PrEP protocol/user-mismatch is pivotal for sustainable HIV prevention. There is an urgent call to increase formal PrEP provision and provide more tailored PrEP care.

## Supporting information

S1 File Interview guide. (DOCX)
