# Supplementary material for: Barriers and missed opportunities in PrEP uptake, use and care among men who have sex with men with recent HIV infection in the Netherlands
Source: PLoS One. 2025 Jan 6;20(1):e0310621. doi: 10.1371/journal.pone.0310621 (PMC11703081; doi:10.1371/journal.pone.0310621)
Supplement: S1 File — (DOCX) [file pone.0310621.s001.docx]

**Interview Guide: PrEP experiences among people living with HIV**

**Introduction**

Welcome, we’re very glad to have you here. First, my colleague and I will introduce ourselves briefly.

- My name is [NAME], and I am a researcher the GGD. I will be leading the interview today.
- My name is [NAME], and I am also a researcher the GGD. I will be listening and may ask additional questions when necessary.

We have invited you to this interview in order to gain insight into your experiences with PrEP. During the course of the interview, personal and intimate topics may be discussed, such as your personal views or sexual experiences. Feel free to say anything you want to share and please do indicate what you don't want to share. There are no wrong answers. The interview aims to be an open conversation in which you can share your experiences while we try to understand them as best as possible. Let us know if you feel that we do not understand or have misunderstand something. Do you have any questions about this so far?

Next, there are some practical things to go through:

- The interview will take about an hour.
- My colleague and I will occasionally take notes during the interview in order to better remember certain aspects of your story.
- Participation in this interview is completely voluntary. You can stop the interview at any time, ask questions or take a break whenever you want to.
- Part of the interview will be recorded. This is necessary to be able to properly understand your story afterwards and to process it in our research. Your privacy as an interviewee is very important here. All recordings and transcripts will be anonymized and we will omit any details that can be traced back to you as a person.
- Before we start the recording, we will first ask for your permission to participate and ask some questions on your personal background.

**Step-by-step plan for the start of the interview**

1. Informed consent

(See attachment)

1. Questions about sociodemographic characteristics

(Answers will be noted down in het table below by the interviewer)

| **Question** | **Answer** |
| --- | --- |
| Age |  |
| Sex  If relevant:   - Sex at birth - Sex change surgery - Gender identity, gender expression |  |
| Country of birth (parents) |  |
| What is the highest level of education you have attained? |  |
| What kind of work do you do? |  |
| Do you have a religion, if so what is it? |  |

1. Turn on recorder and say: “this is interview number ___, and today’s date is __/__/____.”

**Interview question for all participants**

1. Have you ever used PrEP?

If YES 🡪 go to INTERVIEW GUIDE 1

If NO 🡪 go to INTERVIEW GUIDE 2

**Interview guide 1: HIV+ MSM aware of PrEP with PrEP experience**

1. When and where did you first hear about PrEP?
2. What were your considerations about whether or not to start with PrEP?
3. How did you make the decision to start with PrEP?
   - *How did it feel to make this decision?*
4. What steps did you take to get PrEP?
   - *Who played a role in this? (Partner? Healthcare provider? Social network?)*
   - *How did you experience PrEP care?*
   - *Route (informal vs formal [NPP, GP, other])*
   - *Time between ‘attempt to obtain PrEP’ and ‘PrEP intake/start’?*
5. *PrEP care:*
   - *Were you tested for HIV at PrEP intake consultation (T0)?*
   - *If not, when was your last hiv-negative test result? Blood vs self-test?*
6. How did you experience the use of PrEP?
   - *What aspects about using PrEP did you find easy*
   - *What aspects about using PrEP did you find difficult?*
   - *What did you expect from using PrEP?*
   - *In what ways did using PrEP meet the expectations you had?*
   - *How did you use PrEP? (daily, event-driven, other)*
   - *How long did you use PrEP?*
   - *Were you able to take pills according to the regimen you chose?*
7. Can you describe if and in what way PrEP influenced your daily life?
8. Did you deliberately stop taking PrEP?

If YES 🡪 what were your reasons for stopping?

- - *When did you stop taking PrEP?*
  - *How did you feel when you stopped?*
  - *What thoughts did you have when you stopped?*
  - *Did you apply any other HIV prevention strategies? If so, which one(s)?*
  - *Do you have any idea why PrEP was not effective for you?*
  - *Have professionals also given you a reason for this?*
  - *Do you have any idea how you could have been supported better or differently in a way that may have led you to continue using PrEP?*

If NO 🡪 can you describe your PrEP use around the time of your HIV diagnosis?

- *In case ‘having a 1^st^ pos HIV test result’ was the reason to stop PrEP:*
  - *When and where was the HIV-test performed?*
  - *Estimated moment of acquiring HIV?*

Optional:

1. How important was it for you to protect yourself against HIV?
2. In what ways did you protect yourself against HIV?

**Ending the interview**

- Is there anything else you would like to say, share or explain?
- Before we finish, there are a few things I would like to reflect on:

1. How did you experience the interview?
2. Were there any questions that were unclear or too difficult?
3. Do you have any questions for me?
4. Do you have suggestions for improvement regarding the interview? Or for me as an interviewer?

**Thank the participant and stop the recording**

**Interview guide 2: HIV+ MSM aware of PrEP without PrEP experience**

1. When and where did you first hear about PrEP?

- Was this before or after your diagnosis?

If BEFORE 🡪 go to question 2

If AFTER 🡪 go to question 4

1. What were your considerations about whether or not to start with PrEP?
2. How did you make the decision to not start with PrEP?
   - *How did it feel to make this decision?*
3. Do you have any idea why you haven't heard about PrEP before?

- Do you have any suggestions about how the information could have reached you better and sooner?

Optional:

1. How important was it for you to protect yourself against HIV?
2. In what ways did your protect yourself against HIV?

**Ending the interview**

- Is there anything else you would like to say, share or explain?
- Before we finish, there are a few things I would like to reflect on:

1. How did you experience the interview?
2. Were there any questions that were unclear or too difficult?
3. Do you have any questions for me?
4. Do you have suggestions for improvement regarding the interview? Or for me as an interviewer?

**Thank the participant and stop the recording**
